# Antimicrobial Action, Genotoxicity, and Morphological Analysis of Three Calcium Silicate-Based Cements

**DOI:** 10.1155/2022/2155226

**Published:** 2022-05-10

**Authors:** Amjad Abu Hasna, Ana Luisa Theodoro, Larissa Marques Pereira, Lucas de Paula Ramos, Tiago Moreira Bastos Campos, Maisour Ala Rachi, Talal Al-Nahalwi, Luciane Dias de Oliveira, Cláudio Antonio Talge Carvalho

**Affiliations:** ^1^Department of Restorative Dentistry, Endodontics Division, Institute of Science and Technology, São Paulo State University-UNESP, São José dos Campos, São Paulo, Brazil; ^2^Department of Biosciences and Oral Diagnosis, Institute of Science and Technology, São Paulo State University-UNESP, São José dos Campos, São Paulo, Brazil; ^3^Physics Department, Aeronautics Technological Institute (ITA), São José dos Campos, São Paulo, Brazil; ^4^Department of Operative Dentistry, Syrian Private University (S.P.U), Damascus, Syria

## Abstract

This study is aimed at evaluating five mineral oxides (5MO), mineral trioxide aggregate repair high plasticity (MTA HP), and mineral trioxide aggregate (MTA) in relation to the antimicrobial action over *Porphyromonas gingivalis*, *Porphyromonas endodontalis*, *Parvimonas micra*, *Fusobacterium nucleatum*, and *Prevotella intermedia*; the genotoxicity over mouse macrophage (RAW 264.7) and osteoblast (Mg-63) cultures; and the morphological analysis using scanning electron microscopy (SEM) analysis (50 k and ×100 k). Sodium hypochlorite (NaOCl), calcium hydroxide, and saline solution were used as control groups in the different analysis. All data were submitted to a normality test and then analyzed with one-way ANOVA, Tukey, and Kruskal-Wallis and Dunn tests, considering *α* ≤ 0.05 significance level. It was found that over *P. gingivalis* and *P. endodontalis*, there was no a significant difference between the calcium silicate-based cements (CSC) and the control group of saline solution, and only 5MO was similar to the NaOCl group. However, over *P. micra*, all groups were effective and showed a statistically significant difference compared to the saline solution group. Conversely, none of the groups were effective over *F. nucleatum* and *P. intermedia*, except of the NaOCl group. There was a significant difference between 5MO and MTA groups in comparison with NaOCl and MTA HP over osteoblasts and macrophages after 24 hours. SEM images showed small irregular particles interspersed with some elongated needle-like particles and small irregular particles with some larger particles as well as elongated particles. It was concluded that 5MO, MTA, and MTA HP have effective antimicrobial action over *P. micra*. However, only 5MO is effective over *P. gingivalis* and *P. endodontalis*. Besides, 5MO and MTA are not genotoxic over mouse macrophage (RAW 264.7) and osteoblast (Mg-63) cultures.

## 1. Introduction

Five mineral oxides (5MO) (Golden Yatti LLC, Muscat, Oman) is a calcium silicate-based cement (CSC), it was introduced firstly as a pulp capping material [1]. In the literature, there are three case reports evaluated its efficacy in endodontic perforation and open apex sealing [2–4]. However, only one experimental study, in dogs, reported its efficacy as a direct pulp capping material [5]. On the other hand, mineral trioxide aggregate repair high plasticity (MTA HP) (Angelus, Londrina, PR, Brazil) is a new version of the mineral trioxide aggregate (MTA) (Angelus, Londrina, PR, Brazil) [6], the commonly used CSC [7]. MTA HP was released firstly in 2016, with the proposal of CSC with improved physical properties because of a high plasticity [6].

There is a lack of studies in the literature about the biological properties of 5MO and MTA HP. The antimicrobial action of CSC is an extremely relevant factor to be studied as the success of sealing demands aseptic areas [8]. Besides, the biocompatibility guarantees the efficacy of CSC sealing ability without causing damage to the host tissues [9], being one of the basic requirements in the evaluation of an endodontic material [10]. Lastly, the morphological analysis of these CSC powder plays a major role to understand the nature and porosity of the generated tissues [11].

Therefore, the aim of this study was to evaluate 5MO, MTA HP, and MTA in relation to : (I) the antimicrobial action over *Porphyromonas gingivalis*, *Porphyromonas endodontalis*, *Parvimonas micra*, *Fusobacterium nucleatum*, and *Prevotella intermedia*; (II) the genotoxicity over mouse macrophage (RAW 264.7) and osteoblast (Mg-63) cultures; and (III) the morphological analysis. The null hypothesis is that CSCs have no antimicrobial action and are genotoxic.

## 2. Material and Methods

### 2.1. Crystal Violet Assay

Five different inocula of *P. gingivalis* (ATCC 33277), *P. endodontalis* (ATCC 35406), *P. micra* (ATCC 23195), *F. nucleatum* (ATCC 25586), and *P. intermedia* (ATCC 33563) were prepared and standardized at (1 × 10^8^ CFU/mL) in a spectrophotometer (Visible Spectrophotometer V-5000, Shanghai Metash Instruments Co., Ltd., China) in saline solution (NaCl 0.9%) (Eurofarma, São Paulo, SP, Brazil).

Biofilms of each inocula were incubated in 96-well microplates (TPP, Trasadingen, Switzerland) at 37°C for 7 days in anaerobic conditions, with replacement of the culture medium every 48 hours. Later, the biofilm measurement test (crystal violet) was performed. Two independent experiments were carried out, with 5 repetitions each, totaling *n* = 10 for each group.

The groups of this study were as follows: (I) saline solution (negative control group) (Eurofarma, São Paulo, SP, Brazil), (II) 2.5% sodium hypochlorite (NaOCl) (Biodynamics, Ibiporã, PR, Brazil) (positive control group), (III) 5MO (Golden Yatti LLC, Muscat, Oman), (IV) MTA HP (Angelus, Londrina, PR, Brazil), and (V) MTA, white version (Angelus, Londrina, PR, Brazil).

To manipulate the CSCs, a sterilized glass plate and a flexible spatula number 24 were used considering the proportion of (3× powder: 1× liquid) according to the manufacturers' protocol. After 24-hours contact with the groups, 200 *μ*L/well of methanol was added for 20 min to fix the biofilm, then the methanol was removed, and the plates were incubated at 37°C for 24 h. Afterwards, 200 *μ*L/well of 1% crystal violet (V/V) (Synth, Diadema, SP, Brazil) was added for 5 min, the dye was removed, and washes with sterile physiological solution (Eurofarma, São Paulo, SP, Brazil) and 33% acetic acid (V/V) (Synth, Diadema, SP, Brazil) were performed. The plates were read in a microplate reader (BIO-TEK Instruments, Highland Park, Winooski, VT, USA), and the optical densities were converted into the biofilm biomass, using the formula:

%Reduction in biomass = (OD − Treated Group × 100)/(Mean OD Control Group).

### 2.2. Genotoxicity Analysis

In this study, cultures of mouse macrophages (RAW 264.7) (Rio de Janeiro Cell Bank-APABCAM–RJ, Brazil) and osteoblasts (MG-63) (Rio de Janeiro Cell Bank-APABCAM–RJ, Brazil) were used. The cells were grown in Dulbecco's Modified Eagle Medium (DMEM) (LGC Biotechnology, Cotia, Brazil) supplemented with 10% fetal bovine serum (FBS) (Invitrogen, New York, USA), incubated at 37°C, with atmospheric humidity, with 5% CO_2_ using cell culture flasks (TPP, Zollstrasse, Switzerland).

The culture medium was changed every 48 h until a state of subconfluence of the cells was observed, and the cells were transferred to another cell flask. The cells were transferred to a falcon-type tube where they were centrifuged for 5 min at 2000 rpm. To quantify the number of viable cells, the Trypan blue (0.4%, Sigma-Aldrich, St. Louis, MO, USA) exclusion test was performed. The cells were cultivated in 96-well microplates, 200 *μ*l of DMEM medium was added and supplemented with 10% FBS containing 2 × 10^4^ viable cells. These plates were incubated (37°C, 5% CO_2_) for 24 hours for cell adhesion. Then, the supernatant was discarded, and the cells were washed with PBS. The incubation period was 5 min and 24 hours. The number of wells was equal to 10 for all groups.

To prepare CSCs, they were manipulated in a 24-well plate following the manufacturer's proportions and remained in the bottom of the well to set. Ca(OH)_2_ (Biodynamics Chemicals and Pharmaceuticals LTDA, Paraná, Brazil) was added in this test, as a positive control group. Ca(OH)_2_ powder was manipulated using the proportion (1 : 1) (powder: liquid). After the materials have set, each well containing the CSCs was filled with 2 mL of culture medium (DMEM) supplemented with 10% fetal bovine serum, and the plates were incubated at 37°C for 24 h with 5% CO_2_. By this form, a conditioned DMEM was obtained of each material, in which 100 *μ*L per well of the conditioned DMEM of each group was applied.

The NaOCl group was used as a negative control group. To verify the viability of the culture, genotoxicity analysis was performed. After the incubation period, the cells were fixed in 100% methanol for 20 min, followed by staining with 4′,6-diamidino-2-phenylindole (DAPI) (Sigma, Missouri, USA), and the dye was removed after 5 min of contact with the cells and then washed with PBS. Micronuclei analysis was performed using a fluorescence microscope (Leica DFC310FX) (Leica Biosystems, Wetzlar, Tokyo, Japan) at ×40 magnification, evaluating 2,000 cells/well.

### 2.3. Morphological Analysis by Scanning Electron Microscopy

This analysis was performed for powder of each CSC in which a thin layer of powder was dispersed over a polymethylmethacrylate plate mounted on an aluminum stub. All stubs were coated with carbon for electrical conductivity. Samples were visualized by SEM (MIRA3-TESCA, Brno-Kohoutovice, Czech Republic). Images of the different components of the CSC microstructure at different magnifications in electron backscatter mode were captured at magnifications up to ×100 k.

### 2.4. Statistical Analysis

Data were submitted to a normality test. The data that presented a normal distribution were analyzed with one-way ANOVA and Tukey test. The data that did not present a normal distribution were analyzed with Kruskal-Wallis test and complemented by the Dunn test, considering *α* ≤ 0.05 significance level using GraphPad Prism 6 (La Jolla, CA, USA).

## 3. Results

### 3.1. Crystal Violet Assay

**Figure 1:**
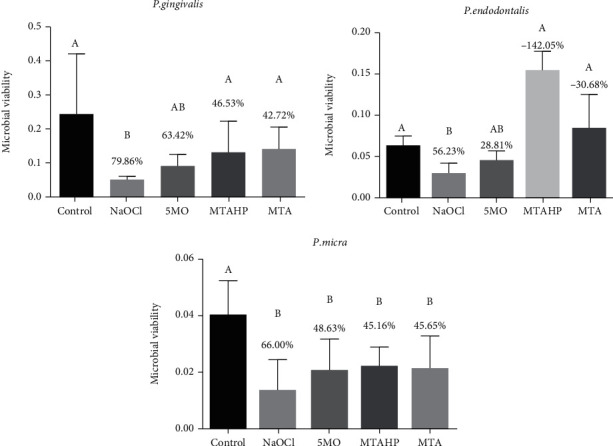
Microbial viability calculated by reflectance and viability reduction (in %) of *P. gingivalis*, *P. endodontalis*, and *P. micra* biofilms by crystal violet assay after treatment with the groups. Different uppercase letters indicate statistical differences.

Over *P. gingivalis* and *P. endodontalis*, there was no a significant difference between the CSCs and the negative control group (saline solution). Only the 5MO group was similar to the NaOCl group. However, over *P. micra*, all groups were effective and showed a statistically significant difference when compared to the negative control group (Figure 1).

**Figure 2:**
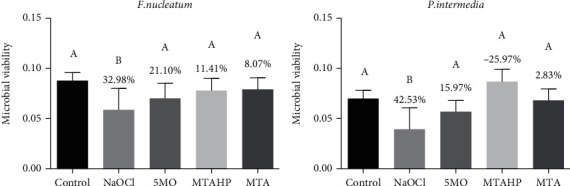
Microbial viability calculated by reflectance and viability reduction (in %) of *F. nucleatum* and *P. intermedia* biofilms by crystal violet assay after treatment with the groups. Different uppercase letters indicate statistical differences.

On the other hand, none of the groups were effective over *F. nucleatum* and *P. intermedia* in which none of the groups showed a significant difference when compared to the saline solution group, except of the NaOCl group (Figure 2).

### 3.2. Genotoxicity Analysis

**Figure 3:**
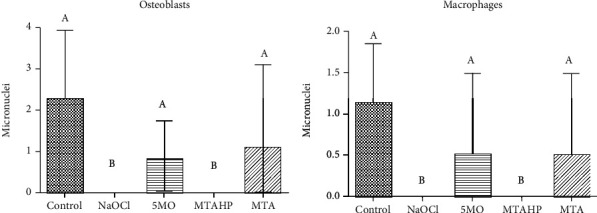
Micronuclei calculated by reflectance of macrophage (RAW 264.7) and osteoblast (Mg-63) cultures after treatment with the groups. Different uppercase letters indicate statistical differences.

There was a significant difference between 5MO and MTA groups in comparison with NaOCl and MTA HP over osteoblasts and macrophages after 24 hours. 5MO and MTA groups were similar to the control group (calcium hydroxide) without a significant statistical difference among them (Figure 3).

### 3.3. Morphological Analysis by Scanning Electron Microscopy

**Figure 4:**
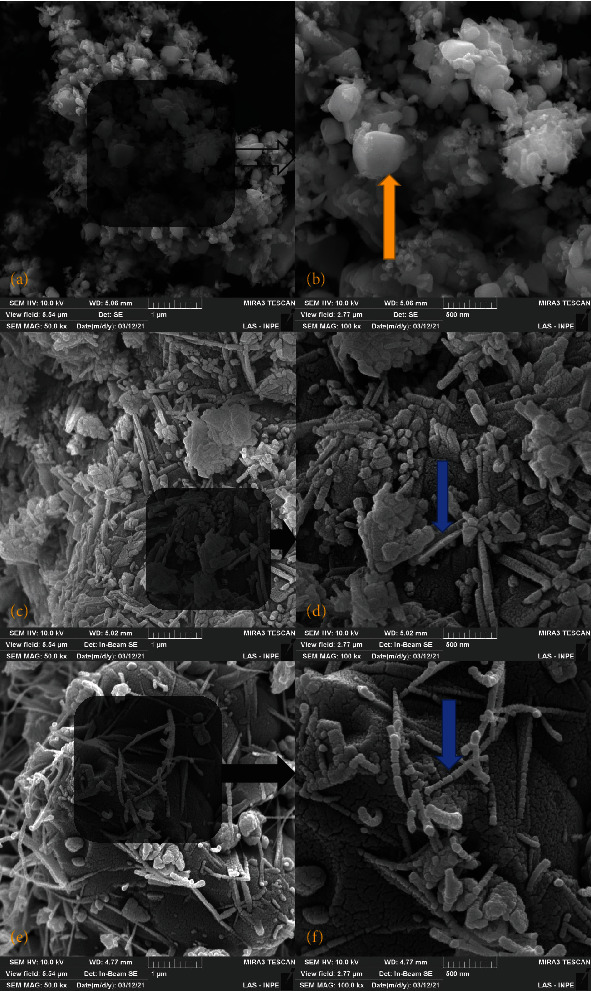
Illustrative images of (a) 5MO powder in ×50 k, (b) 5MO powder in ×100 k, (c) MTA HP powder in ×50 k, (d) MTA HP powder in ×100 k, (e) MTA powder in ×50 k, and (f) MTA powder in ×100 k by scanning electron microscopy. Blue arrow indicates needle-like particles, and golden arrow indicated irregular particles.

SEM images showed small irregular particles interspersed with some elongated needle-like particles and small irregular particles with some larger particles as well as elongated particles (50 k and ×100 k) (Figure 4).

## 4. Discussion

This study was elaborated to evaluate the antimicrobial action, genotoxicity, and the morphological characteristic of three different CSCs including 5MO, MTA HP, and MTA. It was found that these CSCs are effective over some anaerobic bacteria; thus, the null hypothesis was rejected.

In the present study, using crystal violet assay, it was found that MTA and MTA HP were not effective over *P. ginigivalis*; however, 5MO was effective and has a significant difference when compared to the saline solution group. In the literature, the antimicrobial action of MTA over *P. ginigivalis* was tested in different studies, and it was not effective after 24, 48, and 72 hours using the inhibition zone test [12, 13].

Different types of MTA were tested over *F. nucleatum*, *P. gingivalis*, and *P. intermedia* in different studies, and it was concluded that MTA (ProRoot) was not effective over these microorganisms and presented no inhibition zone after 24, 48, and 72 hours [12, 14, 15]. However, it is effective in preventing *F. nucleatum* leakage [16, 17]. In the present study, none of the groups were effective over *F. nucleatum* and *P. intermedia* in which none of the groups showed a significant difference compared to the saline solution group, except of the NaOCl group.

In a more recent study, it was found that MTA HP was not effective over *P. gingivalis* [18], and these outcomes agree with the reported results in the present study, in which MTA HP was not effective over none of the tested anaerobic bacteria except of *P. micra*. Besides, there is only one study in the literature that evaluated the effect of MTA over *P. endodontalis* in which the effectivity of MTA was improved with the addition of nitric oxide [19]. According to the same study, the antimicrobial action of the CSC is affected by its setting [19]. In the present study, only 5MO was effective over *P. endodontalis.*

The endodontic infection is more complex than it was thought. There is an involvement of diverse factors including microorganisms, their byproducts, and growth factors [20]. However, the effective disinfection is not given by the CSCs alone, but by the irrigants, intracanal medications, and the complementary techniques [21–25] highlighting the sealing role of the CSCs [2, 4, 26].

In the literature, it was reported that MTA is not genotoxic when evaluated over mouse lymphoma cells [27], Chinese hamster ovary cells [28], human peripheral lymphocytes [29], murine fibroblasts [30], and human dental pulp stem cells [31] using different concentration of MTA and different evaluation methods, thus, indicating its use as a pulp caping material or a as root canal sealer as in Fillapex MTA [32, 33]. In the present study, MTA was not genotoxic and has a significant difference when compared to the NaOCl group over mouse macrophage (RAW 264.7) and osteoblast (Mg-63) cultures.

To the best of our knowledge, there is no studies in the literature evaluating the genotoxicity of MTA HP and 5MO making the results of the present study pioneers, where 5MO was not genotoxic and has no significant difference when compared to the control group, and MTA HP was genotoxic over mouse macrophage (RAW 264.7) and osteoblast (Mg-63) cultures.

The morphological analysis by SEM showed small irregular particles interspersed with some elongated needle-like particles and small irregular particles with some larger particles as well as elongated particles (50 k and ×100 k) of the tested CSCs in which all these cements favor tissue depositing to from dentin-like tissue bridge [34].

This is an in vitro study; thus, it has limitations to perfectly simulate the clinical conditions. Therefore, clinical studies are indicated to confirm or contradict the outcomes of the present study.

Finally, the CSCs have a finality of promoting tissue repair in interesting areas for apexogensis, apexification, and apicectomy [3–5, 35] or sealing of perforated area [2] and some advantages in vital pulp therapy approaches [36]. Thus, the most expected of these material are not to be genotoxic, and has antimicrobial action, or to not favor the contamination; still, this action may be improved by the use of antimicrobial agents like sodium hypochlorite and others [24, 37, 38] and by complementary technique like photodynamic therapy and passive ultrasonic irrigation [21, 23, 25, 37].

## 5. Conclusions

- 5MO has effective antimicrobial action over *P. gingivalis*, *P. endodontalis*, and *P. micra*
- MTA and MTA HP have effective antimicrobial action over *P. micra*
- 5MO and MTA are not genotoxic over mouse macrophage (RAW 264.7) and osteoblast (Mg-63) cultures

## Data Availability

The data used to support the findings of this study are available from the corresponding author upon request.
